# Antioxidant, Antimicrobial, Anticancer, and Molecular Docking Insights into *Pancratium maritimum* Seeds and Flowers: A Phytochemical Approach

**DOI:** 10.1002/open.202400407

**Published:** 2025-01-10

**Authors:** Muhammed Sait Ertuğrul, Özge Balpınar, Erdi Can Aytar, Betul Aydın, Emine Incilay Torunoglu, Alper Durmaz, Altevir Rossato Viana

**Affiliations:** ^1^ Hemp Research Institute Ondokuz Mayıs University Samsun 55200 Türkiye; ^2^ Faculty of Agriculture Department of Horticulture Usak University Uşak 64200 Türkiye; ^3^ Faculty of Science Department of Biology Gazi University Ankara 06500 Türkiye; ^4^ Faculty of Medicine Department of Medical Biochemistry Necmettin Erbakan University Konya 42090 Türkiye; ^5^ Ali Nihat Gökyiğit Botanical Garden Application and Research Center Artvin Çoruh University 08000 Artvin Türkiye; ^6^ Department of Biochemistry and Molecular Biology Federal University of Santa Maria Santa Maria Brazil

**Keywords:** Antioxidant activity, GC-MS analysis, Molecular docking, *Pancratium maritimum*, STARD10 protein, SW480 cell line

## Abstract

This study investigates the antioxidant, antimicrobial, and anticancer properties of *Pancratium maritimum* L. in Sp. Pl.: 291 (1753) seeds and flowers. Antioxidant activity was assessed using DPPH free radical scavenging and iron chelation assays. Antimicrobial evaluations assessed the efficacy of the extracts against diverse microorganisms. Cell viability assays were conducted on the dukes c colon cancer (SW480), while gas chromatography‐mass spectrometry (GC‐MS) analysis facilitated the identification of bioactive compounds. The ethanol extract of *P. maritimum* seeds exhibited a total phenolic content of 296.89±14.53 mg GAE/g extract DW and a total flavonoid content of 361.03±20.18 mg QE/g extract DW. Conversely, the flower extract showed a total phenolic content of 95.03±7.22 mg GAE/g extract DW and a total flavonoid content of 272.12±16.42 mg QE/g extract DW. As a result, the ethanol extract of *P. maritimum* seeds contains higher phenolic and flavonoid contents than the flower extract. Antimicrobial evaluations demonstrated significant inhibitory effects of both seed and flower extracts, with minimum inhibitory concentration (MIC) values ranging from 25 to >50 mg/mL. Notably, the seed extract showed greater activity against *E. coli* and *C. krusei*. GC‐MS analysis identified 18 bioactive compounds in the seed extract and 16 in the flower extract, with crucial components including ethyl oleate and 5‐hydroxymethylfurfural. Additionally, cell viability assays revealed that ethanol extracts from seeds and flowers significantly reduced SW480 cell viability, particularly at doses of 750 μg/mL and 250 μg/mL, respectively. These findings underscore the therapeutic potential of *P. maritimum* in terms of its antioxidant, antimicrobial, and anticancer properties, highlighting its value as a natural source of antioxidants and antimicrobial agents. Furthermore, the molecular docking study emphasises strong binding interactions of key compounds, particularly ethyl oleate and hexadecanoic acid ethyl ester, with the human STARD10 protein. The biological interactions and health implications of *P. maritimum* provide a significant foundation for future research in drug development and therapeutic applications.

## Graphical abstract

This study explores the antioxidant, antimicrobial, and anticancer properties of *Pancratium maritimum* L. seeds and flowers. Ethanol extracts show high phenolic and flavonoid content, strong antimicrobial activity, and significant cytotoxic effects on SW480 colon cancer cells. GC‐MS analysis identifies bioactive compounds, and molecular docking highlights key interactions with STARD10 protein, underscoring the plant‘s therapeutic potential.

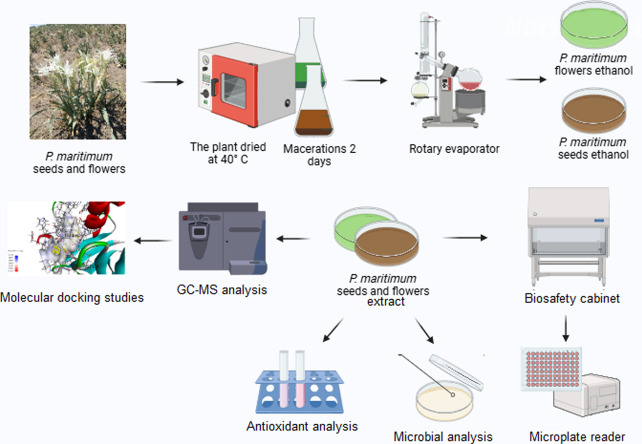

## Introduction

Colorectal cancer is described as the second most common cancer worldwide, with 1.7 million cases diagnosed annually.^[1]^ Despite substantial advancements in treatment approaches and early diagnostic technologies, drug resistance remains a major obstacle in cancer therapy. This challenge underscores the urgent need to discover effective and reliable compounds with minimal side effects, particularly in cases where current therapeutic options are limited, and adverse effects are a concern. Herbal products, extensively utilised in traditional medicine, hold significant potential in preventing and treating various diseases, including cancer. Their richness in bioactive compounds and generally lower side effect profiles have made them a focal point of interest in modern medical research.^[2]^

**Figure 1:**
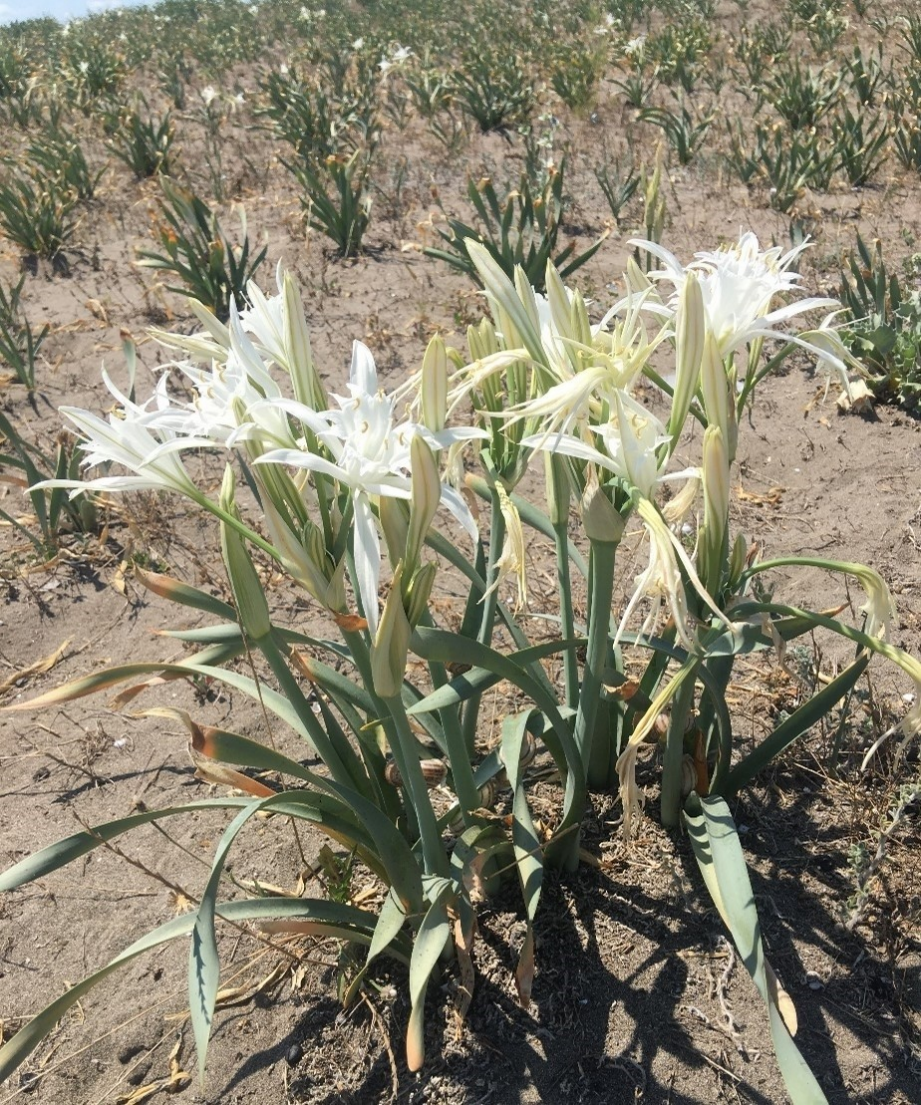
*P. maritimum* plant morphological structure.

*Pancratium maritimum* L. in Sp. Pl.: 291 (1753) belonging to the Amaryllidaceae (J.St.‐Hil.), is one of the 24 species classified within the genus *Pancratium* Dill. ex L., commonly referred to as the sea daffodil. While the genus *Pancratium* comprises 24 species globally, only one species, *P. maritimum*, is naturally distributed in Türkiye (Figure 1).[^3^, ^4^, ^5^] This bulbous and perennial plant has a wide native range, primarily spanning the Canary Islands, the Mediterranean region, and the Black Sea coasts. Its distribution extends from Portugal, Morocco, and the Balearic Islands to Türkiye, Syria, Israel, and the Caucasus. Additionally, it is found along the southern coastline of Bulgaria and the northern shores of Turkey and Georgia. Furthermore, the species has successfully naturalised in certain regions beyond its native range, including southern California, Bermuda, and the Azores, showcasing its ecological adaptability to diverse environmental conditions.^[4]^

*P. maritimum* grows in coastal dunes and beaches, with much of its leaves and flower stems often buried in sand. The plant has a long‐necked bulb and glaucous, broadly linear evergreen leaves, though they often die back in hot summers. Its flower stem can grow up to 40 centimetres tall. The plant produces 3 to 15 white flowers in an umbel, each up to 15 centimetres long. The flower's corona is two‐thirds the length of its tepals. These flowers have a subtle, exotic lily scent most noticeable on calm, windless summer nights. The blooming period is from June to October.[^6^, ^7^]

*P. maritimum* found in Sicily, Italy, exhibits antioxidant properties,^[8]^ In Tunisia, *P. maritimum* demonstrates antioxidant, anticoagulant, biofilm inhibitory, and antibacterial activities.^[9]^ *P. maritimum*, found in India, has anti‐inflammatory properties.^[10]^ Alkaloids isolated from *P. maritimum* in Squillace, Italy, display antiviral activity.^[11]^ The alkaloid Pancratistatin, isolated from *P. maritimum* in Marūḥ, Egypt, exhibits anticancer properties.^[12]^ *P. maritimum* collected in Matrouh, Egypt, shows both anticancer and antimicrobial activities^[13]^ *P. maritimum* grown in Djerba, Tunisia, demonstrates antioxidant and antimicrobial activities.^[9]^

Due to their rich phytochemical content*, P. maritimum* root, leaf, and fruit extracts are expected to control oxidative stress effectively. Antioxidants protect against reactive oxygen species generated by oxidative stress in the body.^[14]^ Antioxidants are chemical substances in various foods, including fruits and vegetables, that help prevent cell damage under various stress conditions.^[15]^ This study aims to evaluate the antioxidant potential of *P. maritimum* under *in vitro* conditions and conduct content analysis to understand which components contribute to this effectiveness. Demonstrating the antioxidant potential of sea daffodil extracts can provide a framework for future studies involving the plant.

Bacterial conditions are one of the determining factors for antimicrobial activity. When exposed to antibiotics, bacterial phenotypes can exhibit various changes; these changes include characteristics such as sensitivity, resistance, tolerance, and persistence.^[16]^ Antimicrobial agets are typically categorised into natural agents and synthetic compounds. Natural antimicrobial agents are derived from living organisms, including curcumin from the Zingiberbuaceae and other substances extracted from filamentous saprophytic microbes or medicinal plants.^[17]^

In this context, the rich compositions of *P. maritimum* indicate significant potential for antimicrobial research. Investigating its antimicrobial properties may support the modern applications of this plant, which has long been used in folk medicine. Thus, the importance of researching to enhance the pharmaceutical value of *P. maritimum* and develop new therapeutic methods to combat microbial resistance becomes even more apparent.

STARD10 is a phospholipid transfer protein that regulates the transport of phosphatidylcholine and phosphatidylethanolamine between intracellular membranes, containing a lipid transfer domain related to the steroidogenic acute regulatory protein (StAR).^[18]^ Erb‐B2 receptor tyrosine kinase 2 (ERBB2), an epidermal growth factor receptor family member, is particularly active in ERBB2‐positive metastatic colorectal cancer patients. The overexpression of ERBB2 leads to an increase in STARD10 expression, which modulates the activity of STARD10 and plays a significant role in cancer progression.^[19]^ Molecular docking studies have revealed that bioactive compounds found in *P. maritimum* seeds, including Ethyl Oleate, Hexadecanoic acid, ethyl ester, and Hordenine, as well as compounds identified in its flowers, such as 5‐Hydroxymethylfurfural, Hexadecanoic acid, 2‐hydroxy‐1‐(hydroxymethyl), and 1,3,5‐Triazine‐2,4,6‐triamine, exhibit strong interactions with the STARD10 protein. These compounds’ binding energies and interaction profiles highlight their potential to modulate STARD10 activity, emphasising its critical role in cancer progression. These findings provide a significant foundation for the development of novel strategies in cancer therapy.

The different parts of *P. maritimum* may exhibit distinct biological activity profiles, which is an important consideration. This study aims to investigate the antimicrobial potential of *P. maritimum* seed and flower extracts and elucidate the plant's role as a natural antimicrobial agent. Limited research has shown that *P. maritimum* possesses anticancer, antiviral, and antioxidant properties, inhibiting cell proliferation in various cancer cell lines, including breast, lung, and prostate. However, further research is needed to explore its medical applications and efficacy, particularly concerning colon cancer, which remains under‐examined. Such investigations could provide valuable insights into the plant's potential anticancer activity.

A molecular docking study has been conducted on the phytochemical compounds identified in the seeds and flowers of *P. maritimum* with the STARD10 protein. There are gaps in the literature regarding this topic. Determining the *in‐silico* interactions of STARD10 proteins with these phytochemical compounds could provide important data on their potential use as therapeutic agents in colorectal cancer treatment.

## Results and Discussions

Our study used various methods to analyse the antioxidant activity and phenolic content of *P. maritimum* seeds and flowers. Our findings are summarised in Table 1. The 2,2‐diphenyl‐1‐picrylhydrazyl (DPPH) IC_50_ value for *P. maritimum* flowers in ethanol was determined to be 0.62±0.01 mg/mL. The DPPH IC_50_ value for the positive control, Butylated hydroxytoluene (BHT), was measured at 0.23±0.01 mg/mL. The iron chelation IC_50_ value for *P. maritimum* flowers was 8.56±0.33 mg/mL, while the Ethylenediaminetetraacetic acid (EDTA) iron chelation IC_50_ value was 5.30±4.44 mg/mL. The total phenolic content (TPC) was 95.03±7.22 mg GAE/g extract DW, and the total flavonoid content (TFC) was 272.12±16.42 mg QE/g extract DW.

**Table 1:** DPPH assay activities of *P. maritimum* seeds and flowers extracts (IC_50_ (mg/mL)±SD) and TPC and TFC±SD* Values (n=3).

Plant Name	DPPH (IC_50_ mg/mL)	Iron Chelating (IC_50_ mg/mL)	TPC (mg GAE/g extract DW)	TFC (mg QE/g extract DW)
*P. maritimum* seeds ethanol	0.45±0.01	8.72±0.20	296.89±14.53	361.03±20.18
*P. maritimum* flowers ethanol	0.62±0.01	8.56±0.33	95.03±7.22	272.12±16.42
BHT (Positive control)	0.23±0,01			
EDTA (Positive control)		5.30±4.44		

In contrast, the DPPH IC_50_ value for *P. maritimum* seeds in ethanol was measured at 0.45±0.01 mg/mL. The DPPH IC_50_ value for the positive control, BHT, was also recorded as 0.23±0.01 mg/mL. The iron chelation IC_50_ value for *P. maritimum* seeds was 8.72±0.20 mg/mL, while the EDTA iron chelation IC_50_ value was 5.30±4.44 mg/mL. The TPC was 296.89±14.53 mg GAE/g extract DW, and the TFC was 361.03±20.18 mg QE/g extract DW. These findings indicate that *P. maritimum* seeds possess superior antioxidant capacity compared to the flowers, associated with their higher total phenolic and flavonoid content.

In this study, the antimicrobial activities of *P. maritimum* seed and flower extracts against various microorganisms were compared with standard drugs such as Ampicillin (Amp), Chloramphenicol (C), and Ketoconazole (Keto). According to the antimicrobial screening results, the minimum inhibitory concentration (MIC) values of *P. maritimum* flowers ranged from 25 to >50 mg/mL against all bacterial and fungal strains. In comparison, Amp exhibited MIC values ranging from 31.25 to >125 mg/mL, C from 15.63 to >125 mg/mL, and Keto from <0.98 to 62.5 mg/mL. Similarly, the MIC values of *P. maritimum* seeds ranged from 25 to >50 mg/mL against all bacterial and fungal strains, with Amp showing MIC values between 31.25 and >125 mg/mL, C between 15.63 and >125 mg/mL, and Keto between <0.98 and 62.5 mg/mL (Table 2). The seed extract exhibits a lower MIC value against the *Escherichia coli* bacterial strain, indicating higher antimicrobial activity. Additionally, the seed extract shows a lower MIC value against the *Candida krusei* fungal strain, demonstrating higher antimicrobial activity.

**Table 2:** MIC, MBC, and MFC of the P*. maritimum* seeds and flowers extracts and controls (mg/ml).

		Plant Species		Positive Control		
Microorganisms		*P. maritimum* flowers	*P. maritimum* seeds	Amp	C	Keto
*E. coli* ATCC 25922	MIC	50	25	>125	125	NS
	MBC	>50	25	>125	>125	NS
*S. aureus* ATCC 25923	MIC	>50	>50	62.5	125	NS
	MBC	>50	>50	62.5	>125	NS
*K. pneumoniae* ATCC 13883	MIC	25	25	125	15.63	NS
	MBC	50	25	125	15.63	NS
*P. vulgaris* RSKK 96029	MIC	50	50	>125	125	NS
	MBC	>50	>50	>125	>125	NS
*B. cereus* NRRL B‐3711	MIC	>50	>50	31.25	125	NS
	MBC	>50	>50	>125	125	NS
*C. albicans* ATCC 10231	MIC	25	25	NS	NS	31.25
	MFC	50	>50	NS	NS	62.5
*C. krusei* ATCC 6258	MIC	50	25	NS	NS	<0.98
	MFC	>50	>50	NS	NS	15.63

Amp: Ampicillin; C: Chloramphenicol; Keto: Ketoconazole; NS: Not Studies.

Various bioactive compounds have been identified in the ethanol extracts of *P. maritimum* seeds and flowers. The extracts’ retention times (RT), concentrations (% area), and chemical structures are presented in Tables 3 and 4. The seed extract contains 18, while the flower extract contains 16 bioactive phytochemical compounds. The main components of *P. maritimum* seeds are Ethyl Oleate (28.51 %), Hexadecanoic acid ethyl ester (palmitic acid) (11.97 %), Hordenine (4.02 %) and in flowers, 5‐Hydroxymethylfurfural (13.44 %), Hexadecanoic acid 2‐hydroxy‐1‐(hydroxymethyl) ethyl ester (Palmitic acid β‐monoglyceride) (5.70 %), 1,3,5‐Triazine‐2,4,6‐triamine (3.15 %), and 4H‐Pyran‐4‐one, 2,3‐dihydro‐3,5‐dihydroxy (2.40 %).

**Table 3:** GC‐MS analysis results of *P. maritimum* seeds.

No	Retention Time (minutes)	Compound Name	Molecular Weight (g/mol)	Base Peak	Area (%)	Structure
1	11.864	Maltol	126.11	126.05	1.02	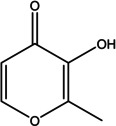
2	19.109	5‐Hydroxymethylfurfural	126.11	97.05	3.89	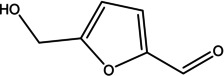
3	31.356	Hordenine	165.23	58.05	4.02	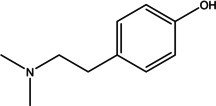
4	31.717	Eicosane	282.5	57.05	1.02	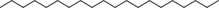
5	31.872	Sucrose	342.30	57.05	1.53	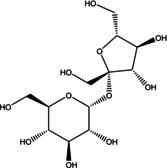
6	48.463	Hexadecanoic acid, ethyl ester	284.5	88.05	11.97	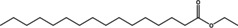
7	50.306	9,12‐Octadecadienoic acid (Z, Z)‐	280.4	67.10	2.07	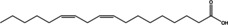
8	50.381	Oleic Acid	282.5	55.10	2.39	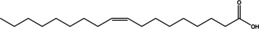
9	50.637	Ethyl Oleate*	310.5	55.05	26.22	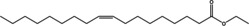
10	50.890	Octadecanoic acid, ethyl ester	312.5	88.10	2.49	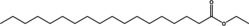
11	51.609	Gamma‐Sitosterol	414.7	225.05	1.69	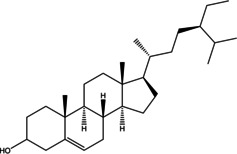
12	51.712	9,10‐Anthracenedione, 1‐amino‐	223.23	223.05	1.55	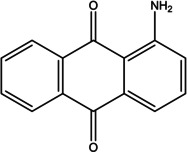
13	53.480	Tetracosamethyl‐ cyclododecasiloxane	889.8	73.05	1.66	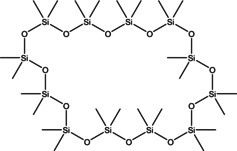
14	53.759	1‐(2,4‐Dihydroxyphenyl)‐3‐ phenyl‐2‐propen‐1‐one	240.25	256.10	1.23	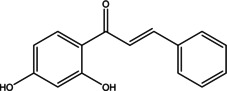
15	54.403	Galathan, 1,2,3,12,15,16‐hexadehydro‐9,10‐ [methylenebis(oxy)]‐	251.28	250.00	1.97	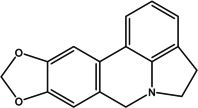
16	54.602	Hexadecanoic acid, 2‐hydroxy‐1‐ (hydroxymethyl)ethyl ester	330.5	98.10	3.54	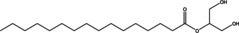
17	54.707	5,6‐Dihydro‐5,6‐dihydroxy‐ 7‐hydroxymethyl‐12‐ methylbenz(a)anthracene, trans‐	306.4	257.10	1.19	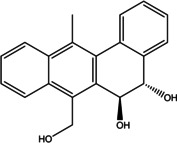
18	56.067	7,12‐Dimethyl‐8,9,10,11‐ tetrahydrobenz(a)anthracene	260.4	248.05	1.28	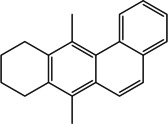

**Table 4:** GC‐MS analysis results of *P. maritimum* flowers.

No	Retention Time (minutes)	Compound Name	Molecular Weight (g/mol)	Base Peak	Area (%)	Structure
1	3.285	Dihydroxyacetone	90.08	31.00	1.54	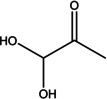
2	12.139	1,3,5‐Triazine‐ 2,4,6‐triamine	126.12	126.05	3.15	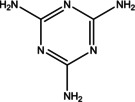
3	13.925	4H‐Pyran‐4‐one, 2,3‐dihydro‐3,5‐ dihydroxy‐6‐methyl‐	144.12	43.00	2.40	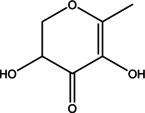
4	19.359	5‐Hydroxymethylfurfural	126.11	97.00	13.44	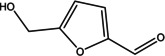
5	19.954	1,2,3‐Propanetriol, 1‐acetate	134.13	43.00	1.49	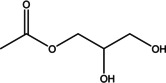
6	23.395	1‐Amino‐4‐ methylpiperazine	115.18	42.00	1.63	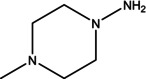
7	24.470	Cyclohexasiloxane, dodecamethyl‐	444.92	340.90	1.00	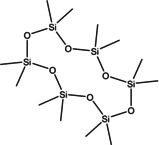
8	27.033	1‐Pentadecene	210.40	43.05	1.06	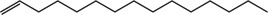
9	48.046	n‐Hexadecanoic acid	256.42	88.00	2.88	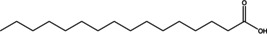
10	48.430	Hexadecanoic acid, ethyl ester	284.5	43.00	1.72	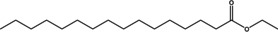
11	49.343	Gamma‐Sitosterol	414.7	42.00	1.88	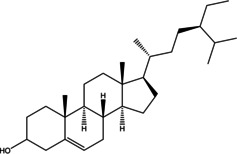
12	50.252	9,12‐Octadecadienoic acid (Z,Z)‐	280.4	255.10	1.55	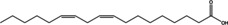
14	50.602	9,12,15‐Octadecatrienoic acid, ethyl ester	306.5	126.05	6.26	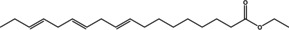
15	54.613	Hexadecanoic acid, 2‐hydroxy‐1‐ (hydroxymethyl)ethyl ester*	330.5	98.10	6.90	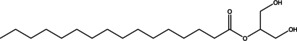
16	55.202	Diisooctyl phthalate	390.6	149.05	1.57	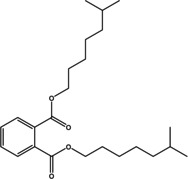

**Figure 2:**
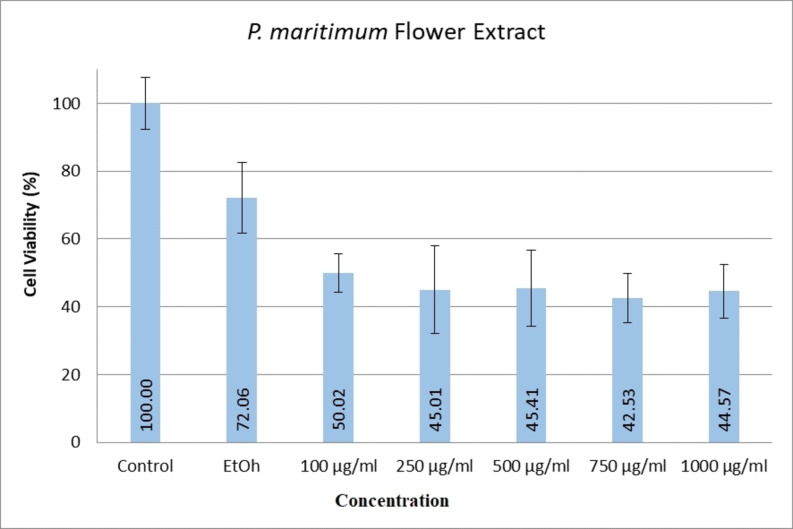
Cytotoxic effects of *P. maritimum* flower extract on SW480 cell line.

**Figure 3:**
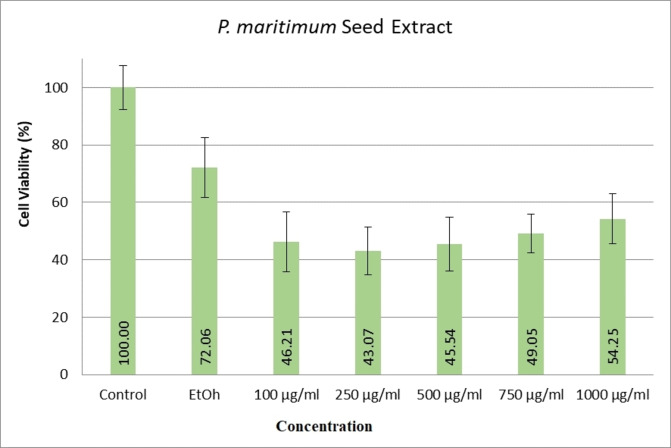
Cytotoxic effects of *P. maritimum* seed extract on SW480 cell line.

When examining the effects of ethanol extracts of *P. maritimum* flowers on human colon cancer (SW480) cell line viability levels, it was determined that all doses significantly reduced cell viability compared to the control and ethanol groups (Figure 2). A similar trend was observed with ethanol extracts of the seeds of the same plant (Figure 3). According to the data, the dose showing the most significant decrease in cell viability was 750 μg/mL for flower extract and 250 μg/mL for seed extract.

**Figure 4:**
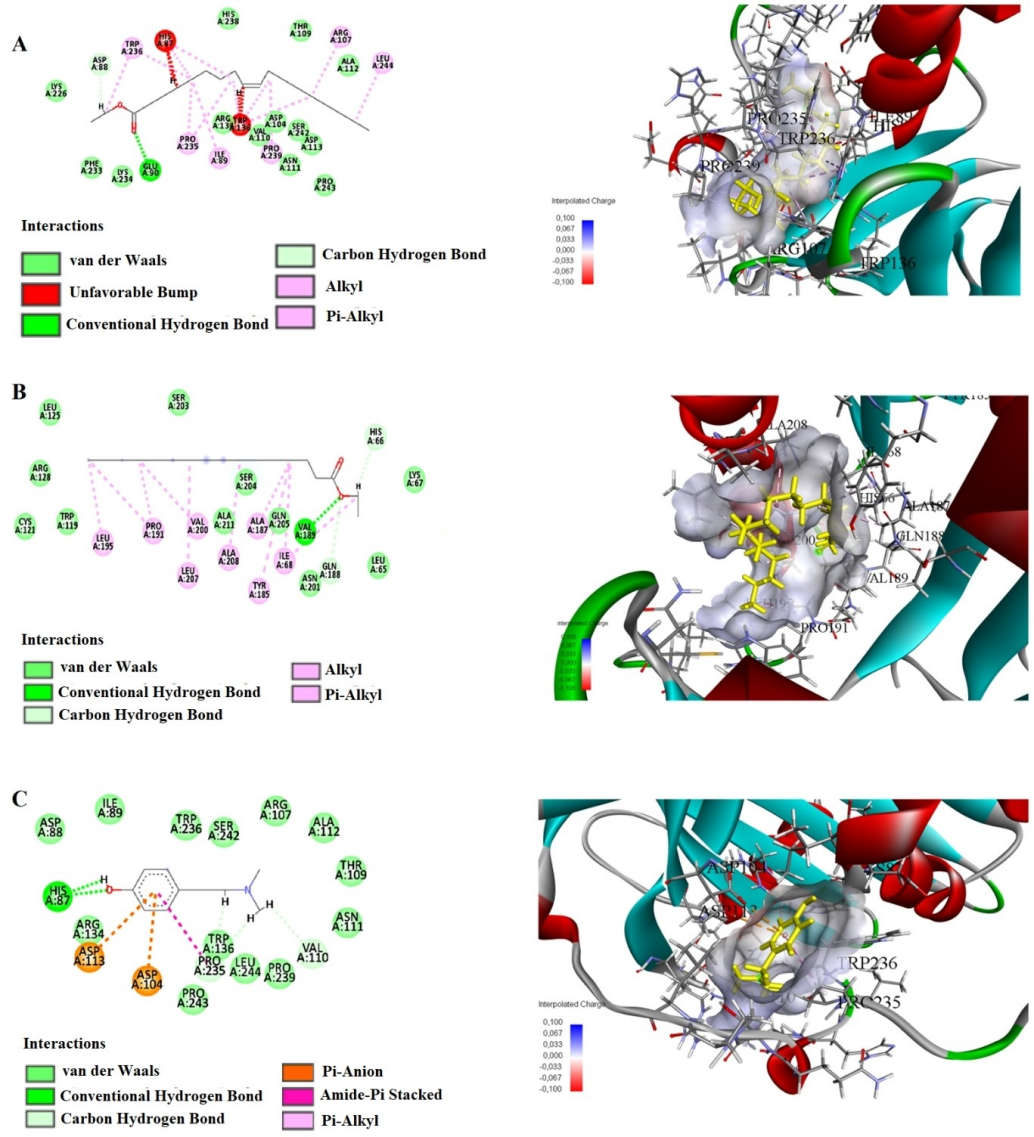
Molecular docking process of A) Ethyl Oleate B) Hexadecanoic acid, ethyl ester C) Hordenin with Human STARD10 (6SER).

**Figure 5:**
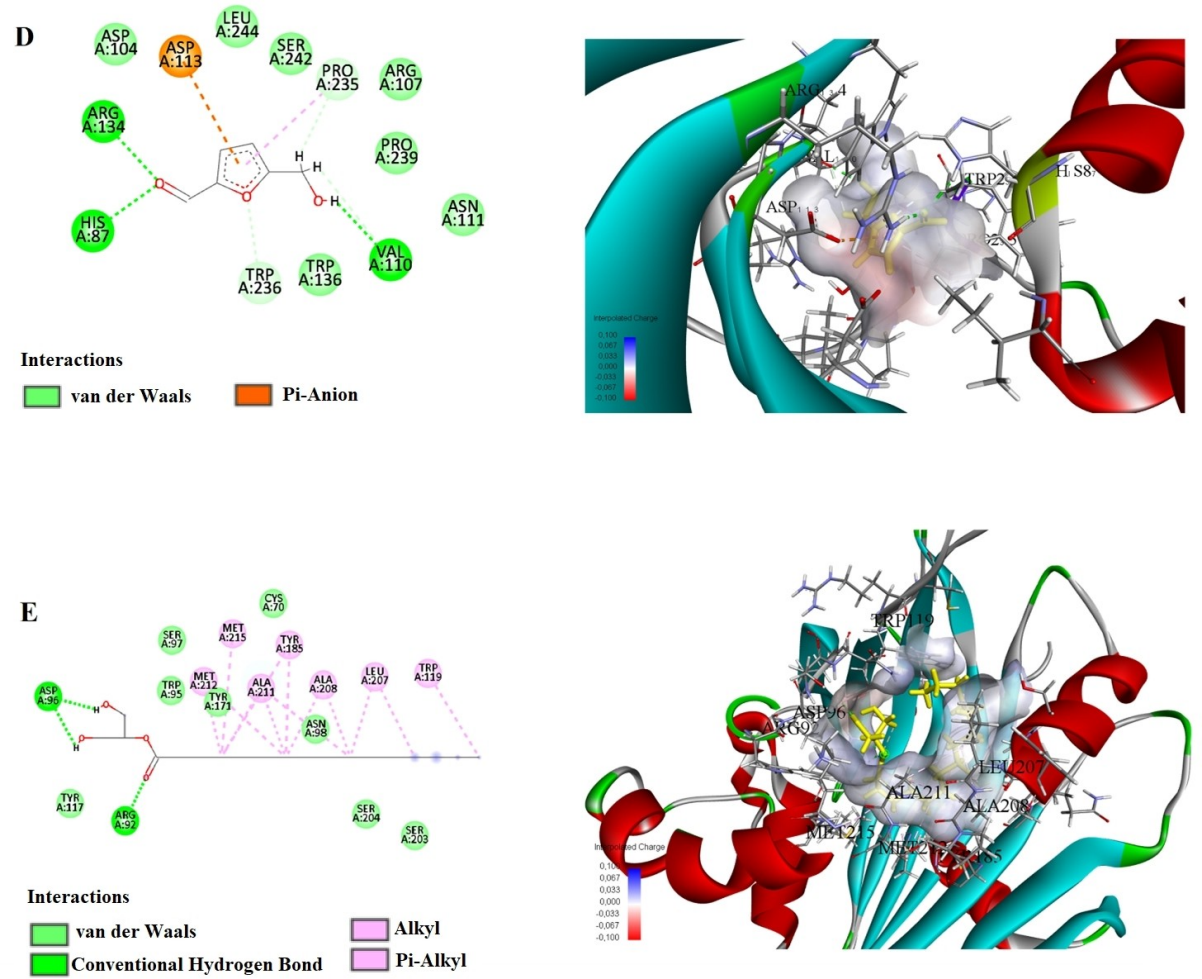
Molecular docking process of D) 5‐Hydroxymethylfurfural E) Hexadecanoic acid, 2‐hydroxy‐1‐(hydroxymethyl) with Human STARD10 (6SER).

**Figure 6:**
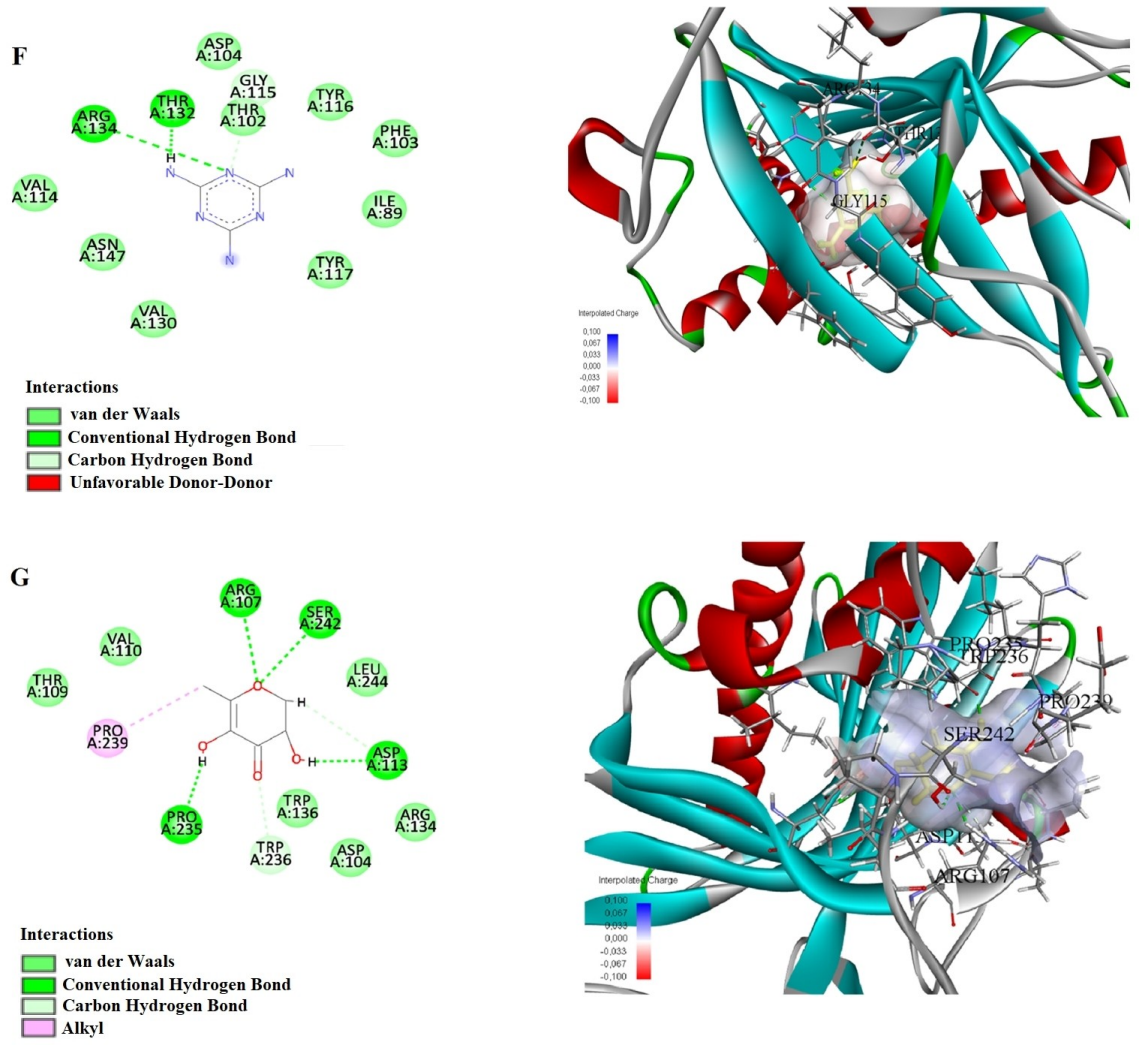
Molecular docking process of F) 1,3,5‐Triazine‐2,4,6‐triamine G) 4H‐Pyran‐4‐one, 2,3‐dihydro‐3,5‐dihydroxy‐with Human STARD10 (6SER).

The results of our molecular docking study evaluated the interactions and binding energies of various organic compounds with specific amino acids. The findings help us understand potential biological interactions and binding properties. In our study, the simulation of 2D and 3D interactions between molecules and the human STARD10 enzyme in a computer environment is shown in Figures 4, 5 and 6. The interactions between the compounds and the enzymes in the best position are shown in Tables 5 and 6.

**Table 5:** Molecular docking energy and report of predicted interactions of compounds from *P. maritimum* seeds against human STARD10 protein (6SER).

Component	Binding Energy (kcal/mol)	Amino acid	Interacting	Distance
Ethyl Oleate	−4.0	A: GLU90 : HN: O1	Conventional Hydrogen Bond	2.17
		H8 ‐ A: ASP88 : OD2	Carbon Hydrogen Bond	2.92
		A: ARG107	Alkyl	4.02
		A:PRO235	Alkyl	5.33
		A:PRO239	Alkyl	4.24
Hexadecanoic acid, ethyl ester	−5.4	A: VAL189 : HN: O2	Conventional Hydrogen Bond	1.91
		A: GLN188: HA: O2	Carbon Hydrogen Bond	2.60
		H5 ‐ A: HIS66 : O	Carbon Hydrogen Bond	1.96
		A: ALA187	Alkyl	4.56
		A:PRO191	Alkyl	5.35
		A: VAL200	Alkyl	4.67
		A: ALA208	Alkyl	4.34
		C1‐ A:PRO191	Alkyl	4.02
		C1‐ A: LEU195	Alkyl	4.98
		A: LEU207	Alkyl	4.59
		A: ILE68	Alkyl	4.30
		C17‐ A: ILE68	Alkyl	4.37
		A: TYR185	Alkyl	5.08
Hordenine	−5.5	A: HIS87 : HD1: O1	Conventional Hydrogen Bond	1.68
		H15‐ A: HIS87 : O	Conventional Hydrogen Bond	1.72
		H4‐ A: VAL110 : O	Carbon Hydrogen Bond	2.07
		H6‐ A:PRO235 : O	Carbon Hydrogen Bond	2.37
		H8‐ A:PRO235 : O	Carbon Hydrogen Bond	2.30
		A: ASP104 : OD2	Pi‐Anion	3.80

**Table 6:** Docking energy and report of predicted interactions of compounds from *P. maritimum* flowers against human STARD10 protein (6SER).

Component	Binding Energy (kcal/mol)	Amino acid	Interacting	Distance
5‐Hydroxymethylfurfural	−5.0	A: HIS87 : HD1: O1	Conventional Hydrogen Bond	2.07
		A: ARG134 : HH11	Conventional Hydrogen Bond	2.11
		H4 ‐ A: VAL110 : O	Conventional Hydrogen Bond	2.14
		A: TRP236: HA: O1	Carbon Hydrogen Bond	2.48
		H5 ‐ A:PRO235 : O	Carbon Hydrogen Bond	3.09
		H6 ‐ A: VAL110 : O	Carbon Hydrogen Bond	3.02
		A: ASP113 : OD2	Pi‐Anion	4.24
		A:PRO235	Pi‐Alkyl	4.93
Hexadecanoic acid, 2‐hydroxy‐1‐(hydroxymethyl)	−5.2	A: ARG92 : HH12: O2	Conventional Hydrogen Bond	1.85
		A: ARG92 : HH22: O1	Conventional Hydrogen Bond	2.27
		H1 ‐ A: ASP96 : OD1	Conventional Hydrogen Bond	2.24
		H4‐ A: ASP96 : OD2	Conventional Hydrogen Bond	1.92
		A: ALA208	Alkyl	4.82
		A: ALA211	Alkyl	4.83
		A: LEU207	Alkyl	5.11
		A: MET212	Alkyl	5.37
		A: MET215	Alkyl	4.84
		A: TRP119:C1	Pi‐Alkyl	4.29
		A: TYR185	Pi‐Alkyl	4.36
1,3,5‐Triazine‐2,4,6‐triamine	−5.0	A: ARG134 : HH21: N3	Conventional Hydrogen Bond	2.73
		H2‐ A: THR132 : O	Conventional Hydrogen Bond	2.95
		A: GLY115 : HA1: N3	Carbon Hydrogen Bond	2.20
4H‐Pyran‐4‐one, 2,3‐dihydro‐3,5‐dihydroxy‐	−5.4	A: ARG107: HE: O1	Conventional Hydrogen Bond	2.53
		A: SER242: HG: O1	Conventional Hydrogen Bond	2.37
		H1‐ A: ASP113 : OD2	Conventional Hydrogen Bond	1.80
		H2‐ A:PRO235 : O	Conventional Hydrogen Bond	1.76
		A: ARG107 : HD2: O1	Carbon Hydrogen Bond	2.47
		A: TRP236: HA: O3	Carbon Hydrogen Bond	2.30
		H8‐ A: ASP113 : OD2	Carbon Hydrogen Bond	2.96
		C6‐ A:PRO239	Alkyl	4.45

This study presents the results of molecular docking analyses of three different compounds (Ethyl Oleate, Hexadecanoic acid ethyl ester, and Hordenine) identified through gas chromatography‐mass spectrometry (GC‐MS) analysis of *P. maritumum* seed extract against human STARD10 protein. The binding energies obtained indicate strong interactions between these compounds and STARD10 protein, as evidenced by their negative values (Ethyl Oleate: −4.0 kcal/mol, Hexadecanoic acid ethyl ester: −5.4 kcal/mol, Hordenine: −5.5 kcal/mol). Ethyl Oleate forms a traditional hydrogen bond between the NH group of GLU90 amino acid and the O1 atom (2.17 Å), as well as a carbon‐hydrogen bond between the OD2 atom of ASP88 and H8 (2.92 Å). Additionally, alkyl interactions were observed with ARG107, PRO235, and PRO239 (4.02 Å, 5.33 Å, and 4.24 Å, respectively). Hexadecanoic acid ethyl ester uses a traditional hydrogen bond with VAL189 : HN: O2 (1.91 Å) and a carbon‐hydrogen bond with GLN188: HA: O2 (2.60 Å). Furthermore, alkyl interactions were noted with HIS66, ALA187, PRO191, VAL200, ALA208, PRO191, LEU195, LEU207, ILE68, and TYR185 (ranging from 1.96 Å to 5.35 Å). Hordenine forms traditional hydrogen bonds with HIS87 : HD1: O1 and HIS87 (1.68 Å and 1.72 Å, respectively), as well as carbon‐hydrogen bonds with VAL110, PRO235, and PRO235 (ranging from 2.07 Å to 2.37 Å). Additionally, a pi‐anion interaction was observed with ASP104 (3.80 Å). These findings underscore the robust binding capabilities of these compounds to the STARD10 protein, as elucidated through molecular docking methodologies. This approach provides valuable insights into the potential biological impacts of these interactions, which are relevant for further exploration in drug design and therapeutic strategies.

This study demonstrates that the active compounds present in the *P. maritumum* flower extract, specifically 5‐Hydroxymethylfurfural, Hexadecanoic acid 2‐hydroxy‐1‐(hydroxymethyl), 1,3,5‐Triazine‐2,4,6‐triamine, and 4H‐Pyran‐4‐one 2,3‐dihydro‐3,5‐dihydroxy, exhibit high binding affinities with the STARD10 protein. Molecular docking analyses reveal that each compound forms stable interactions with STARD10, with binding energies of −5.0 kcal/mol, −5.2 kcal/mol, −5.0 kcal/mol, and −5.4 kcal/mol, respectively. 5‐Hydroxymethylfurfural establishes conventional hydrogen bonds with amino acids such as HIS87, ARG134, and VAL110, and forms carbon‐hydrogen bonds with TRP236 and PRO235. Additionally, interactions involving pi‐anion with ASP113 and pi‐alkyl with PRO235 are observed. Hexadecanoic acid, 2‐hydroxy‐1‐(hydroxymethyl), forms conventional hydrogen bonds with ARG92, ASP96, and other amino acids, and exhibits alkyl interactions with ALA208, ALA211, LEU207, MET212, MET215, and pi‐alkyl interactions with TRP119 and TYR185. 1,3,5‐Triazine‐2,4,6‐triamine forms conventional hydrogen bonds with ARG134, THR132, and GLY115, and establishes a carbon‐hydrogen bond with TRP236. Lastly, 4H‐Pyran‐4‐one, 2,3‐dihydro‐3,5‐dihydroxy‐, forms conventional hydrogen bonds with ARG107, SER242, and ASP113, and engages in carbon‐hydrogen bonds with PRO235, ARG107, TRP236, and other amino acids. Also, alkyl interactions with PRO239 and at the C6 position with PRO239 are observed. These findings underscore the potential interactions of compounds isolated from *P. maritimum* seeds and flower extracts with the STARD10 protein, elucidating their molecular mechanisms. This study highlights the successful application of molecular docking methods in understanding these interactions, providing a foundational basis for drug design, and elucidating biological effects.

When we conducted a literature review on *P. maritimum*, we found limited studies, particularly noting the absence of research on iron chelation activity related to *P. maritimum* and its species. Microbial studies are notably sparse based on the literature review. Additionally, limited research concerning antioxidant phenolic contents is associated with *P. maritimum*. Our study reveals that *P. maritimum* exhibits excellent antioxidant and antimicrobial effects. Plants with high antioxidant and phenolic contents are considered to have potential benefits in cancer prevention and treatment by neutralising free radicals and reducing cellular damage, thus potentially inhibiting cancer development. Therefore, plants rich in antioxidants and phenolic compounds are believed to hold significant promise in cancer research.

In the study by Melliti et al.^[9]^ the TPC of the above ground methanolic extract of *P. maritimum* has been reported as 161.31±3.9 mg GAE/g DW, while the TFC is 126.7±3 mg QE/g DW. According to the study by Melliti et al. the methanol extract obtained from the aerial parts of *P. maritimum* has a higher phenolic content than the ethanol extract of *P. maritimum* flowers and a lower phenolic content than the seeds. Additionally, compared to the methanol extract from the aerial parts of *P. maritimum*, the total phenolic and flavonoid contents of the ethanol extracts from the seeds and flowers of *P. maritimum* are higher.

In the study by Leporini et al.^[20]^ demonstrated that the ethanol extract of *P. maritimum* flowers had a TPC of 228.6±2.4 mg GAE/g extract DW and a TFC of 45.7±0.2 mg QE/g extract DW. The ethanol extract of *P. maritimum* fruits had a TPC of 277.8±2.9 mg GAE/g extract DW and a TFC of 52.7±0.3 mg QE/g extract DW. The ethanol extract of *P. maritimum* bulbs had a TPC of 48.4±1.5 mg GAE/g extract DW and a TFC of 24.4±0.3 mg QE/g extract DW. Additionally, it was reported that the ethanol extracts of *P. maritimum* flowers, fruits, and stems exhibited IC_50_ values of 81.3±8.1 μg/mL, 846.1±44.2 μg/mL, and 84.1±8.4 μg/mL, respectively, in the DPPH test. According to the study by Leporini et al., the total phenolic content of the ethanol extract from *P. maritimum* flowers is higher than ours, while the TFC is also higher.

Similarly, when comparing the TPC of the ethanol extract from *P. maritimum* fruits in Leporini et al. study with our findings, the TPC of the ethanol extract from *P. maritimum* flowers in our study is lower, but the TFC is higher. Additionally, compared to the TPC of the ethanol extract from *P. maritimum* bulbs in Leporini et al. study, the TPC and TFC of the ethanol extract from *P. maritimum* flowers in our study are higher. In the study by Leporini et al. the TPC and TFC of the ethanol extracts from *P. maritimum* flowers, bulbs, and fruits are lower than the methanol extracts from *P. maritimum* flowers and seeds in our study. In the study by Leporini et al. the IC_50_ values of *P. maritimum* flowers and stems in the DPPH test indicate that the antioxidant activity of the ethanol extracts from *P. maritimum* flowers and seeds is high compared to our findings. Additionally, Leporini et al. reported that the antioxidant activity of *P. maritimum* fruit extract is lower than that of seed and flower extracts.

In the study by Nikolova et al.^[21]^ determined that the IC_50_ value for *P. maritimum* methanol bulb extract is more significant than 200 μg/mL. In the study by Nikolova et al. it was observed that the methanol extracts of *P. maritimum* flowers and seeds exhibited good antioxidant activity compared to the methanol extract of *P. maritimum* bulbs.

In the study by Taie et al.^[22]^ the TPC and TFC of *P. maritimum* leaf extract were measured at 5.36 mg GAE/g extract DW and 1.17 mg QE/g extract DW, respectively. Furthermore, the methanol extracts of *P. maritimum* flowers and leaves demonstrated inhibition rates of 85.2 % and 81.3 %, respectively. Taie et al. found that, compared to our study, the TPC and TFC of the ethanol extracts from *P. maritimum* flowers and seeds were higher than those of the *P. maritimum* leaf extract. Additionally, it was observed that both the methanol extracts of *P. maritimum* flowers and seeds exhibited good antioxidant activity compared to the methanol extracts of *P. maritimum* flowers and leaves.

In the study by Melliti et al.^[9]^ the MIC of the essential oil of *P. maritimum* was found to range from 3.25 to 26 mg/mL, whereas the MIC for the microemulsion ranged from 1.625 to 26 mg/mL. Notably, the microemulsion exhibited superior antibacterial activity against the tested bacterial strains compared to the essential oil. The antibacterial effect was particularly pronounced against Gram‐positive bacteria, with significant activity observed against *E. faecalis*, demonstrating MIC and minimum bactericidal concentration (MBC) values of 1.625 mg/mL and 3.25 mg/mL, respectively. The highest recorded MIC value was 3.25 mg/mL for *S. aureus* and *E. faecalis*. Additionally, while the essential oil of *P. maritimum* showed considerable antibacterial activity against all strains tested, it exhibited a relatively weak potential against *Candida glabrata*, with an MIC value of 13 mg/mL.

In contrast, the microemulsion of *P. maritimum* demonstrated enhanced efficacy against *Candida albicans* and *Candida parapsilosis*, with recorded MIC values of 0.4 mg/mL. In our study, compared to the work conducted by Melliti et al. the *P. maritimum* flower and seed extracts demonstrate lower antimicrobial and antifungal activities against the essential oil and microemulsion of *P. maritimum*. These findings underscore the varying efficacy of *P. maritimum* extracts and formulations against different microorganisms, highlighting the importance of formulation strategies, such as microemulsion, in enhancing antimicrobial activity.

In the study by Melliti et al.^[9]^ the methanolic extract of *P. maritimum* demonstrated an MIC of 10 mg/mL against *C. albicans* ATCC 942. The extract also showed MIC values of 20 mg/mL against *Staphylococcus aureus* ATCC 6583, 40 mg/mL against *Pseudomonas aeruginosa* ATCC 27583, and 10 mg/mL against *E. coli* ATCC 25922. Our study found that the ethanol extract of *P. maritimum* flowers and seeds exhibited good antifungal activity against the *C. albicans* ATCC 10231 strain. In contrast, the study by Melliti et al. indicated that the methanol extract of *P. maritimum* demonstrated stronger antifungal activity. Additionally, the ethanol extract of *P. maritimum* flowers and seeds showed effective antimicrobial activity against the *S. aureus* ATCC 25923 strain. In contrast, the methanol extract in the study by Melliti et al. also displayed strong antimicrobial activity against this strain.

The study by Hetta et al.^[13]^ reported that the fruit ethanol extract of *P. maritimum* exhibits significant inhibition effects on *Trichophyton mentagrophytes* and *Aspergillus fumigatus*. It is particularly effective against *C. glabrata*. The fruit ethanol extract also shows effective inhibition against *Streptococcus pneumoniae*. Moreover, it demonstrates pronounced inhibition effects on *Klebsiella pneumoniae*. These findings underscore the strong antimicrobial properties of *P. maritimum* fruit ethanol extract against various microorganisms. The *P. maritimum* seed extract exhibits higher antimicrobial activity against the *E. coli* bacterial strain in our study. The *P. maritimum* seed extract also demonstrates higher antimicrobial activity against the *K. pneumoniae* bacterial strain. Furthermore, the seed extract also shows higher antifungal activity against the *C. krusei* fungal strain.

In the study Bonvicini et al.^[23]^ according to the antimicrobial screening results, the MIC values of *Pancratium illyricum* bulbs ranged from 19.5 to 156 mg/mL against all bacterial and fungal strains. At the same time, Amp exhibited MIC values ranging from 0.078 to 1.25 mg/mL. The seed extract showed a lower MIC value against the *E. coli* bacterial strain, indicating higher antimicrobial activity. In our study, compared to the work of Bonvicini et al., the *P. maritimum* flower and seed extracts exhibit lower antimicrobial and antifungal activities against *P. illyricum* bulbs.

Determining the TPC in plant extracts is directly linked to the antioxidant capacity of these bioactive compounds. They can act as reducing agents, free radical scavengers, metal chelators, or deactivators of singlet oxygen.^[20]^ Oxidative stress can lead to damage in cell structure, potentially contributing to neoplastic transformation and playing a significant role in cancer initiation and progression.^[24]^ In this context, combating oxidative stress with potent antioxidant agents is a prominent area of research. Studies have investigated the antioxidant activity of *P. maritimum* extracts using various *in vitro* methods, demonstrating significant antioxidant effects that are concentration dependent.

In a study by Leporini et al. on *P. maritimum*, extracts from the plant's stem, flowers, bulbs, and fruits were utilised. It was found that direct ethanol extraction from flowers had higher total phenol and total flavonoid content compared to extractions using ether, ethanol, and water, respectively. Antiproliferative activity studies on flower ethanol extracts were conducted using human breast cancer (MCF‐7), human cervical cancer (HeLa), human breast cancer (MDA‐MB‐231), human melanoma (C32), human lung carcinoma (A549), human prostate cancer (LNCaP, and PC3) cell lines, with IC_50_ values ranging from 85.5±3.5 to 176.7±3.9 μg/mL. Flower extracts exhibited antioxidant activity according to DPPH, 2,2′‐Azino‐bis (3‐ethylbenzothiazoline‐6‐sulfonic acid (ABTS), β‐carotene bleaching, and FRAP tests.^[20]^ Furthermore, Youssef et al. purified pancratistatin from *P. maritimum* flower ethanol extracts and applied it to MDA‐MB‐231, HeLa, human colorectal cancer (HCT116), and human dermal fibroblast (NHDF) cell lines, demonstrating selective action against cancer cell lines.^[12]^ McLachlan et al. investigated the cellular mechanisms of pancratistatin, revealing its disruption of mitochondrial membrane potential, increase in caspase 3 and proteasome activities, and augmentation of mitochondrial ROS production.^[25]^ Additionally, GC‐MS analysis detected Hydroxymethylfurfural in plant flowers. In a study by Chow et al. Hydroxymethylfurfural was reported to affect aquaporin‐1 on the HT29 cell line, altering ion conductivity and inhibiting cell migration.^[26]^ Using ethanol extracts *of P. maritimum* flowers at 100–1000 μg/mL doses, similar effects were observed on SW480 cell line viability in this study. Moreover, findings indicated that cell viability approached approximately 50 % compared to the control group across all application doses.

No information was found in the literature regarding the anticancer effects of *P. maritimum* seed extracts. According to the data, seed ethanol extracts contain 28.51 % Ethyl Oleate, 11.97 % Hexadecanoic acid ethyl ester (palmitic acid), and 1.01 % Hordenine. Studies suggest that palmitic acid induces endoplasmic reticulum (ER) stress, calcium release from the ER, and transferrin‐dependent ferroptosis. However, palmitic acid alone does not affect Cluster of Differentiation (CD36) expression, indicating its lack of sole efficacy.^[27]^ An investigation with oleic acid reported increased apoptotic cell numbers in the human colon adenocarcinoma (HT‐29) cell line at 48 and 72 hours,^[28]^ yet another study found oleic acid to have mitogenic effects on cancer cells.^[29]^ Additionally, Anwar et al. identified Hordenine's high binding to pyruvate dehydrogenase kinase‐3 (PDK3) in A549 and human lung carcinoma (H1299) cell lines, inhibiting glycolysis metabolism and affecting cellular energy cycles.^[30]^ Considering these findings, seed extracts appear to be as effective as flower extracts, likely due to the synergistic effects of their constituent compounds.

Maltol is a compound known for its anticancer^[31]^ and antioxidant^[32]^ properties. 5‐Hydroxymethylfurfural exhibits antioxidant and anticancer activities,^[33]^ as well as antimicrobial activity.^[34]^ Hordenine exhibits antimicrobial,^[35]^ antioxidant,^[36]^ and anticancer^[30]^ activities. Eicosane exhibits antifungal^[37]^ activity. Sucrose demonstrates antimicrobial^[38]^ and antioxidant^[39]^ activities. Hexadecanoic acid, ethyl ester, has been reported to possess antioxidant and antimicrobial activities.^[40]^ 9,12‐Octadecadienoic acid (Z, Z) has been reported to possess antioxidant and anticancer activities.^[41]^ Additionally, 9,12‐Octadecadienoic acid (Z, Z) possesses antimicrobial activity.[^42^, ^43^] Oleic acid has been reported to exhibit antibacterial, antifungal, antioxidant, and anticancer activities.^[44]^ Ethyl oleate exhibits antioxidant and antibacterial activity.^[45]^ 9‐octadecenoic acid, methyl ester, possesses antioxidant and anticancer activity.^[46]^ Gamma‐sitosterol exhibits antibacterial, anticancer, antifungal and antioxidant activity.^[47]^ 9,10‐Anthracenedione, 1‐amino, has been reported to possess antimicrobial, anticancer, antioxidant, and cytotoxic activities.^[48]^ Hexadecanoic acid, 2‐hydroxy‐1‐(hydroxymethyl) ethyl ester exhibits antioxidant activity.^[49]^

Dihydroxyacetone is an antifungal agent.^[50]^ 1,3,5‐Triazine‐2,4,6‐triamine exhibits antimicrobial, antioxidant, and anticancer properties.[^51^, ^52^] 4H‐Pyran‐4‐one, 2,3‐dihydro‐3,5‐dihydroxy‐6‐methyl exhibits antioxidant activity.^[53]^ 1‐Amino‐4‐methylpiperazine has been found to exhibit antioxidant, anticancer, and antimicrobial activities.[^54^, ^55^, ^56^] n‐Hexadecanoic Acid exhibits antioxidant, antimicrobial, and anticancer properties.[^57^, ^58^] 9,12,15‐Octadecatrienoic acid, ethyl ester antioxidant and anticancer properties.^[59]^ Diisooctyl phthalate has been reported to exhibit antioxidant, antibacterial, and antifungal effects.^[60]^

*P. maritimum* seed and flower extracts have been traditionally used in medicine and are known for their diverse biomedical effects. These effects include antiviral, antimicrobial, immunostimulant, analgesic, antimalarial, antitumor, antifungal, and antioxidant activities. Additionally, the plant has been reported to play significant roles in the treatment of neurological disorders such as Alzheimer's and Parkinson's diseases.^[61]^ In conclusion, the antioxidant, anticancer, and antimicrobial activities observed in *P. maritimum* seed and flower extracts are thought to be attributed to their rich phytochemical content. These phytochemicals include compounds such as Maltol, 5‐Hydroxymethylfurfural, Hordenine, Eicosane, Sucrose, Hexadecanoic acid ethyl ester, 9,12‐Octadecadienoic acid (Z, Z), Oleic acid, Ethyl oleate, Gamma‐sitosterol, 9,10‐Anthracenedione, 1‐Amino, 1,3,5‐triazine‐2,4,6‐triamine, 4H‐Pyran‐4‐one, 2,3‐dihydro‐3,5‐dihydroxy‐6‐methyl, 1‐Amino‐4‐methylpiperazine, and Diisooctyl phthalate. These compounds have been reported in various studies to exhibit significant antioxidant, antimicrobial, and anticancer activities, which likely contribute to the observed biological effects of the extracts. The bioactive compounds identified through GC‐MS profiling hold potential as therapeutic agents for the treatment of colon cancer and could also serve as valuable candidates for rational drug design.

## Conclusions

This study highlights the therapeutic potential of *P. maritimum*, a member of the Amaryllidaceae, by focusing on its antioxidant, antimicrobial, and anticancer activities. Ethanol extracts of *P. maritimum* seeds and flowers demonstrated strong antioxidant activity, as determined by DPPH assays and total phenolic and flavonoid content measurements. The high levels of phenolic and flavonoid compounds suggest that these extracts can effectively combat oxidative stress by neutralising reactive oxygen species. Antimicrobial tests revealed that *P. maritimum* extracts exhibit significant antimicrobial properties against various bacterial and fungal strains. Specifically, the seed extracts showed higher antimicrobial activity against *E. coli* and *C. krusei*, as indicated by lower MIC values. This indicates the potential use of *P. maritimum* extracts as natural antimicrobial agents. Ethanol extracts of *P. maritimum* significantly reduced the viability of SW480 colon cancer cells. The extracts induced a dose‐dependent decrease in cell viability, with the most pronounced effects observed at concentrations of 750 μg/mL for the flower extract and 250 μg/mL for the seed extract. This suggests that *P. maritimum* extracts have potential anticancer properties, particularly against colorectal cancer. Molecular docking studies revealed that bioactive compounds *in P. maritimum* extracts exhibit strong interactions with the human STARD10 protein. The binding energies and interaction profiles suggest that these compounds can modulate the activity of STARD10, which plays a significant role in cancer progression. This provides a molecular basis for the anticancer effects observed in cell culture studies. The findings of this study underscore the potential of *P. maritimum* as a source of natural bioactive compounds for therapeutic use.

## Materials and Methods

**Figure 7:**
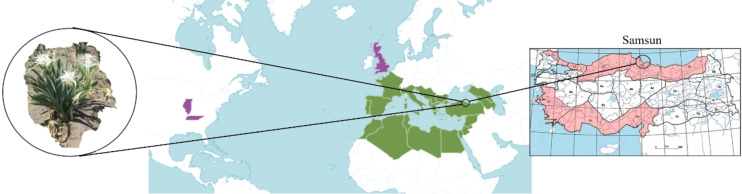
Map image of the collection site of the plant in *P. maritimum*.

**Collection of plant material**. The plant materials of *P. maritimum* used in the study were collected from homogeneous populations on the coastal dunes of Samsun province, particularly in the Costal and Hürriyet areas of the Çarşamba district, the beach dunes in front of the Kızılay camp, the beach dunes of the 19 Mayıs district and the Kızılırmak delta, as well as the beach dunes of Alaçam and Bafra (Figure 7). The sampling was conducted in September‐October. Dr. Alper DURMAZ identified the plant samples that were collected. The *P. maritimum* samples are registered in the Herbarium of the Department of Biology at Ondokuz Mayıs University (OMUB) under the accession number OMUB‐8363.

**Plant extraction**. The flowers and seeds of *P. maritimum* were dried at 40 °C in an oven and ground into a powder using a blender. Extraction was performed using the maceration method recommended by Aytar and Aydın .^[62]^ The aboveground parts were extracted with ethanol at room temperature in a dark environment for two days. The obtained ethanol extracts were filtered through filter paper. Following filtration, the solvent was evaporated under reduced pressure at 40 °C using a rotary evaporator, and the solid extracts were stored at 4 °C.

An appropriate extraction method is crucial for obtaining high yields of bioactive compounds and enhancing antioxidant activity. Extraction methods and processing parameters directly influence the extract's yield, composition, and activity. Non‐toxic solvents are preferred for plant extractions; while water is effective for extracting polar compounds, organic solvents may provide better results for less polar compounds. Although methanol is effective for extracting phenolic compounds among organic solvents, it raises safety concerns. Ethanol, with its lower toxicity and safety for pharmaceutical use, is preferred. Ethanol extracts, particularly in antioxidant assays like DPPH, have demonstrated superior performance, indicating a higher antioxidant potential than other solvents.^[63]^

**Gas chromatography‐mass spectrometry analysis**. GC‐MS analysis was conducted by the methodology outlined by Aytar and Aydın.^[62]^ The analysis utilised a SHIMADZU GCMS‐QP2010 Mass Spectrometer and an AOC‐5000 Auto‐Injector. An Rxi‐5MS column (30 m×0.25 mm×0.25 μm) was employed with a 30–450 Da scanning range. The electron ionisation system was set to 70 eV ionisation energy, and helium gas was used at a constant flow rate of 1 ml/min. The injection volume was 1.5 μl with a split ratio 10 : 1. The injector temperature was maintained at 250 °C, while the ion source temperature was set at 200 °C. The oven temperature was initially held at 70 °C for 10 minutes, then gradually increased at a rate of 3 °C/min to 150 °C, which was maintained for 5 minutes. Subsequently, the oven temperature was raised by 10 °C/min to 250 °C, where it was held for an additional 5 minutes. The solvent delay ranged from 0 to 2 minutes, and the total GC/MS run time was 56.67 minutes. For the liquid sampling method, orchid samples extracted with methanol were diluted 100 times and placed in 1.5 ml vials. The available NIST 2017 databases were used for this chemical analysis.

### Spectroscopic Analysis of Secondary Metabolites

**Total phenolic content**. The TPC was quantified using a modified version of the method described by Singleton and Rossi.^[64]^ In this process, the extract with a concentration of 1 mg/mL was diluted with distilled water in a 1 : 1 ratio, and 200 μL of Folin‐Ciocalteu reagent was added to achieve a homogeneous mixture. This mixture was incubated at room temperature for 3 minutes, after which 1 mL of 2 % sodium carbonate solution was added to stop the reaction, and it was subsequently incubated in the dark for an additional hour. The absorbance of the resulting solution was measured at 765 nm using an ultraviolet (UV) spectrophotometer (Thermo Scientific Varioskan). The results were expressed as milligrams of gallic acid equivalents per gram of dry weight extract (mg GAE/g extract DW), and all measurements were repeated in triplicate.

**Total flavonoid content**. Some modifications were made based on the method of by Osuna‐Ruiz et al.^[65]^ The extract's TFC was determined using the AlCl_3_ method. Firstly, 1 mL of extract (1 mg/mL) was diluted with 6.4 mL of distilled water. Subsequently, 0.3 mL of NaNO_2_ (5 %) was added, and the mixture was allowed to stand for 5 minutes. Next, 0.3 mL of AlCl_3_ (10 %) was added, and the solution was incubated for 6 minutes. In the second step, 2 mL of NaOH (1 M) was added, and the solution was allowed to stand at room temperature for 30 minutes. The absorbance of the resulting solution was measured at 510 nm using a UV spectrophotometer. The TFC was expressed as milligrams of quercetin equivalents per gram of dry weight extract (mg QE/g extract DW). All measurements were repeated three times.

### Determination of Antioxidant Capacity

**DPPH assay**. Modifications were made based on the methodology of Braca et al.^[66]^ focusing on reducing DPPH radical (0.6 mmol stock) in an alcoholic solution facilitated by an antioxidant acting as a hydrogen donor. The absorbance for the blank was recorded at 0 minutes using 1 ml of DPPH and 2 ml of ethanol without sample addition.

The DPPH free radical scavenging activity was determined through a series of steps: DPPH (8 mg) was dissolved in ethanol (100 ml) to achieve a concentration of 80 μg/ml. Stock solutions of plant extracts were prepared at 1 mg/ml, followed by serial dilutions. Each solution (2 ml) was mixed with 2 ml of DPPH solution and incubated in the dark at room temperature for 30 minutes.

The reaction of antioxidants with DPPH, in the presence of a hydrogen donor, resulted in the conversion to the DPPH‐H form, causing a decrease in absorbance and a colour change indicative of electron capture. The absorbance was measured at 517 nm using a UV spectrophotometer. DPPH scavenging activity (%) was calculated using the formula:

DPPHscavengingactivity(%inhibition)=[(A_control-A_sample)/A_control]×100

A concentration curve was plotted to determine the extract concentration that would cause a 50 % reduction in initial DPPH concentration, and the IC_50_ value was obtained via linear regression analysis. A lower I_C50_ value signifies a stronger antioxidant capacity. BHT was used as a reference standard, with all measurements conducted in triplicate. The results are expressed in the format of (IC_50_ mg/mL).

**Determination of ferrous ion chelating capacity**. The extract's ferr ous ion chelating capacity was measured based on the method of Dinis et al.^[67]^ Varying concentrations of the extract were mixed with 135 μL of the solvent. 2 mM FeCl_2_ was added to the solution and incubated for 5 min. After that, five mM ferrozine solution was added and lasted 10 min. After incubation, absorbance was measured at 562 nm by using a spectrophotometer (Thermo Scientific Varioskan Flash) against a blank.

The calculation was performed using the following formula:

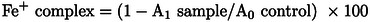




(A_0_: OD of FeCl_2_ and Ferrozine solution without extract or standard A_1_: OD of FeCl_2_ and Ferrozine solution with extract or standard)

EDTA was used as a reference standard. All measurements were performed in triplicate. The results are expressed in the format of (IC_50_ mg/mL).

**Determination of Antimicrobial Activity**. *Gram‐positive strains S. aureus* ATCC 25923, *B. cereus* NRRL B‐3711, Gram‐negative strains *K. pneumoniae* ATCC 13883, Proteus vulgaris RSKK 96029, and *E. coli* ATCC 25922 yeast strains *C. albicans* ATCC 10231 and *C. krusei* ATCC 6258 strain was cultured in sterile Sabouraud Dextrose Broth.

The antimicrobial activity of *P. maritimum* extract against bacteria and yeast strains was screened by microdilution method according to recommendations of clinical and laboratory standards institute (CLSI) reference methods for bacteria with M07‐A7,^[68]^ and for fungi with M27‐A3.^[69]^ MIC, MBC, and minimum fungicidal concentrations (MFC) were determined by using 96‐well plates, and the analysis was carried out in triplicate.^[70]^ Amp and C were positive controls for bacteria and yeast, respectively.

**Cell culture studies**. SW480 cell line was obtained from ATCC. Experiments were conducted at the Laboratory of Cannabis Research Institute, Ondokuz Mayıs University. Cells stored at −80 °C were thawed at room temperature, centrifuged, and the supernatant was discarded. Cells were then incubated in flasks at 37 °C with 5 % CO2 in Dulbecco's Modified Eagle Medium (DMEM) growth medium supplemented with 10 % Fetal Bovine Serum (FBS) and 1 % antibiotics (penicillin, streptomycin, and amphotericin B). Upon reaching sufficient cell density, cells were passaged and seeded into 96‐well plates for further incubation.^[71]^ When wells reached 80 % confluence, extracts were applied at specified doses (100, 250, 500, 750, and 1000 μg/mL). After 24 hours of treatment, MTT (2,3‐bis‐(2‐methoxy‐4‐nitro‐5‐sulfophenyl)‐2H‐tetrazolium‐5‐carboxanilide) reagent was added to the wells and incubated for 4 hours at 37 °C in the dark. Formazan crystals were dissolved in 100 μL Dimetilsülfoksit (DMSO), and spectrophotometric measurement was performed at 570 nm (reference wavelength 630 nm) to determine cell viability.^[72]^

**Molecular docking studies**. Molecular docking is an essential computational tool in drug design that employs algorithms to predict the binding affinities and interactions of ligands such as luteolin, cynaroside, and isoorientin with target proteins. This method contributes to identifying potential drug candidates, optimising molecular structures, and understanding complex molecular recognition mechanisms critical for effective pharmaceutical design.

The protein (PDB ID: 6SER) was downloaded from the Protein Data Bank (PDB) and prepared by removing water molecules and heteroatoms and adding polar hydrogens using BIOVIA Discovery Studio Visualizer 2021. After selecting the embedded ligand, a binding site grid was generated using the “Define and Edit Binding Site – From Current Selection” tool in BIOVIA Discovery Studio Visualizer 2021. The protein was saved in.pdb format and converted to. pdbqt format using AutoDock Tools (v‐1.5.7). The internal ligand was extracted from the grid box, pasted into a new window, and saved in.pdb format. This ligand was also converted to. pdbqt format using AutoDock Tools (v‐1.5.7). The drawn ligand underwent energy minimisation, was saved in.pdb format, and converted to. pdbqt format using AutoDock Tools (v‐1.5.7). Docking scores were obtained for specific poses and utilised for scoring analysis from the initial pose. Log and output files were generated.^[73]^ BIOVIA Discovery Studio Visualizer 2021 analysed amino acid interactions.^[74]^

**Statistical analyses**. Correlation coefficients (R) were calculated using the CORREL function in MS Excel to assess the relationship between two variables. Data are presented as mean±SD from three independent observations, with analysis conducted using SPSS 21.

## Author Contributions

EİT Writing – manuscript, visualisation, research. ECA Analysis, writing – manuscript, data analysis, research, experiments, interpretation. BA Experiments, data analysis, interpretation, research. AD Material collection and extraction. ÖB Experiments, data analysis, interpretation. MSE Experiments, data analysis. ARV Article review

## Conflict of Interests

The authors declare that no conflicts of interest exist.

1

## Data Availability

The datasets used and/or analysed during the current study are available from the corresponding author upon reasonable request.
